# The co-chaperone p23 controls root development through the modulation of auxin distribution in the *Arabidopsis* root meristem

**DOI:** 10.1093/jxb/erv330

**Published:** 2015-07-10

**Authors:** Stefano D’Alessandro, Serena Golin, Christian S. Hardtke, Fiorella Lo Schiavo, Michela Zottini

**Affiliations:** ^1^Department of Biology, University of Padova, Via U. Bassi 58/B, I-35131 Padova, Italy; ^2^University of Lausanne - Biophore Building, DBMV CH-1015 Lausanne, Switzerland

**Keywords:** *Arabidopsis*, auxin, p23-chaperone, pin formed protein (PIN), polar auxin transport, root growth.

## Abstract

Homologues of the p23 co-chaperone of HSP90 are present in all eukaryotes, suggesting conserved functions for this protein throughout evolution. Although p23 has been extensively studied in animal systems, little is known about its function in plants. In the present study, the functional characterization of the two isoforms of p23 in *Arabidopsis thaliana* is reported, suggesting a key role of p23 in the regulation of root development. *Arabidopsis* p23 mutants, for either form, show a short root length phenotype with a reduced meristem length. In the root meristem a low auxin level associated with a smaller auxin gradient was observed. A decrease in the expression levels of PIN FORMED PROTEIN (PIN)1, PIN3, and PIN7, contextually to an inefficient polar localization of PIN1, was detected. Collectively these results suggest that both *Arabidopsis* p23 isoforms are required for root growth, in particular in the maintenance of the root meristem, where the proteins are located.

## Statement

p23 co-chaperones play a key role in the root meristem maintenance via regulation of auxin signalling and the consequent balance between cell differentiation and division rate at the transition zone.

## Introduction

Root growth and development are fundamental processes for the life of plants, controlling water uptake, nutrient acquisition, anchorage to soil, and secondary metabolite synthesis and storage (Saini *et al.*, 2013). Post-embryonic growth and development are controlled through the meristems, which generate differentiating cells that shape adult plant structures (Moubayidin *et al.*, 2009). Meristem maintenance is controlled through a finely regulated orchestration of hormones and signalling molecules, among which auxins are indeed the master regulators of root development (Saini *et al.*, 2013). Auxin is virtually involved in all phases of plant growth and development, and auxin signalling is regulated at the level of biosynthesis, transport, and perception (Saini *et al.*, 2013). From the sites of biosynthesis (Ljung *et al.*, 2005), auxin is actively transported via the combination of two routes involving the long-distance transport through mature phloem and cell-to-cell polar transport (PAT), mediated through specific influx/efflux carriers (Vieten *et al.*, 2007; Geisler *et al.*, 2014). AUX1 and LAX (LIKE-AUX1) are major auxin importers, whereas PIN (PIN FORMED PROTEIN) and ABCB (ATP-BINDING CASSETTE B) are the main families of auxin exporters (Geisler and Murphy, 2006; Verrier *et al.*, 2008; Krecek *et al.*, 2009). At the site of action, auxin perception is mediated through members of the TIR1 (TRANSPORT INHIBITOR RESPONSE 1)/AFB family. TIR1 is an F-box subunit of the ubiquitin ligase complex SCF^TIR1^, which interacts with and ubiquitinates Aux/IAA proteins (AUXIN/INDOLE-3-ACETIC ACID) (Gray *et al.*, 2001). Aux/IAA proteins are auxin response inhibitors that interact and block ARF (AUXIN RESPONSE FACTOR) transcription factors (Mockaitis and Estelle, 2008). Indole-3-acetic acid (IAA) is the most abundant natural auxin, and the apoplastic acidic environment facilitates the protonation of IAA to IAAH, which diffuses into cells. Due to the chemical nature of auxins, PIN and ABCB exporters are the primary control elements of PAT (Geisler and Murphy, 2006). Auxin signalling continuously crosses pathways with other hormones and signalling molecules in a complex chain of feedback controls (Dello Ioio *et al.*, 2008; Overvoorde *et al.*, 2010; Saini *et al.*, 2013). In particular, auxins and cytokinins are the major players of a well-known crosstalk mechanism that regulates root meristem maintenance and consequent root growth (Moubayidin *et al.*, 2009). Cytokinin–auxin crosstalk occurs at the transport level of auxin signalling through a regulatory circuit converging on the type-B ARR1 (ARABIDOPSIS RESPONSE REGULATOR 1) transcription factor and consequently on the SHY2 (SHORT HYPOCHOTHYL 2) protein, which negatively regulates the expression of PIN genes (Dello Ioio *et al.*, 2008).

The small acidic protein, p23, has been identified in animal systems as an HSP90 co-chaperone (Johnson and Toft, 1994), which stabilizes the active conformation of the progesterone-receptor HSP90 complex after binding to the ATP-bound form of HSP90 and lowering the ATPase activity rate of HSP90 (Chadli *et al.*, 2000; Ali *et al.*, 2006; Li *et al.*, 2012). Two homologues of p23 have been identified in *Arabidopsis*: p23-1 (At4g02450) is 241 amino acids in length with a mass of 25.47kDa, whereas p23-2 (At3g03773) is 150 amino acids in length with a mass of 17.4kDa (Zhang *et al.*, 2010). Each isoform shares 27% and 25% amino acid identities with human p23, respectively, and shows 38–60% identity with other plant p23 homologues. The difference in length of p23-1 and p23-2 reflects a glycine rich (MG/GA) segment of 70 amino acids in the C-terminal region of p23-1, whose function is not yet understood. Both plant p23 isoforms and the chimeric protein p23-1-d (lacking the glycine rich tail) bind to HSP90 *in vitro*; however, unlike their animal counterpart, these proteins do not reduce the chaperone ATPase activity rate (Zhang *et al.*, 2010).

Although *Arabidopsis* p23 isoforms have been the object of previous biochemical characterization studies (Zhang *et al.*, 2010; Tosoni *et al.*, 2011), the physiological role of these proteins remains elusive. These analyses revealed that p23 proteins are chiefly located in the root meristem, where they intervene in the regulation of root growth and development.

## Material and methods

### Plant materials and growth conditions

All experiments were performed using *Arabidopsis thaliana* ecotype Columbia (Col-0). The mutant seedlings for *p23-1* (SAIL 245_H06), *p23-2* (SALK_003076), and *arr1-4* (SALK_042196) were obtained from the European *Arabidopsis* Stock Centre (NASC). T-DNA insertions were verified through PCR (primers are listed in Supplementary Table S1 available at *JXB* online). From the p23 single mutants, a double mutant *p23-1*×*p23-2* (*dKO*) was generated. The plant seeds were surface sterilized in 70% EtOH and 0.05% Triton X-100, followed by 100% EtOH. The seeds were sown onto square Petri dishes containing one-half-MS medium supplemented with 0.5g/l MES-KOH, pH 5.7, 0.8% Plant Agar (Duchefa), and 1% Sucrose, stratified for 2 d at 4 °C in the dark, and placed vertically in a growth chamber under a long daylight period (16h light/ 8h dark) using 150 μmol m^−2^ s^−1^ at 22 °C. The translational reporter plants, pp23-1:p23-1-GUS and pp23-2:p23-2-GUS, were generated after transforming the single mutants *p23-1* or *p23-2* with *Agrobacterium* harbouring a construct containing the entire sequence of the *p23-1* or *p23-2* genes, starting from base pair −540 or −3191, respectively, fused to the β-Glucuronidase reporter gene (*UID*-A; the primers are reported in Supplementary Table S1) and subcloned into the pGreen 0029 binary vector (Hellens *et al.*, 2000). Complemented lines p23-1 T 35S:p23-1 and p23-2.1 T 35S:p23-2 were generated after transforming the single mutants *p23-1* or *p23-2* with *Agrobacterium* harbouring a construct containing the coding sequence (CDS) of p23-1 or p23-2 under the control of the CaMV 35S constitutive promoter (primers for CDS cloning are reported in Supplementary Table S1) and subcloned into the pGreen 0029 binary vector (Hellens *et al.*, 2000). The reporter lines DR5:GUS, DII-Venus, PIN1-GFP, PIN7-GFP, or the mutant line *arr1-4*, were crossed with the *dKO* line, and the F2 homozygous lines were used for subsequent analyses.

### RNA isolation and qRT-PCR

Seven days after germination (dag), the roots and shoots of wild-type and mutant seedlings were harvested separately for subsequent analyses. Total RNA was extracted from plant samples using TRIzol® Reagent (Invitrogen) according to the manufacturer’s instructions. First-strand cDNA synthesis was performed using 1 μg of RNA, oligo(dT) primers, and SuperScript-II Reverse Transcriptase (Invitrogen) according to the manufacturer’s instructions. The primers for qRT-PCR (quantitative real-time PCR) are shown in Supplementary Table S1. The expression levels of each gene were normalized to the expression level of the housekeeping genes *Actin-2* (ACT2; At3g18780) or *elf1α* (At5g60390), as indicated.

### Root growth assay

The plant seeds from the different genotypes were sown onto solid growth medium, and vertically grown as described above. Plates were scanned using a flatbed scanner every day from the fourth to the 10th dag. The roots were analysed at different developmental stages through image analysis (Fiji – ImageJ bundle software). The experiments were performed at least three times and each sample comprised 25 seedlings.

For N-1-naphtylphtalamic acid (NPA) and auxin treatments, 5-d-old seedlings were transferred to growth medium supplemented with different concentrations of NPA, IAA, or 1-Naphthaleneacetic acid (NAA; Sigma-Aldrich). After 5 d of treatment, the primary root length was measured as described above.

### β-Glucuronidase histochemical assay

Histochemical staining was performed at different developmental stages. Samples were analysed for β-glucuronidase activity. The samples were incubated for variable times (1h to overnight) at 37 °C in the reaction medium (2mM X-Gluc, 0.05% Triton X-100, 5mM K_3_Fe(CN)_6_, 5mM K_4_Fe(CN)_6_ 3× H_2_O, 10mM EDTA, and 50mM sodium phosphate buffer, pH 7.0). The seedlings were mounted on chloral hydrate solution. The images were captured using a microscope (Leica 5000B) with Normarsky correction (DIC). The experiments were performed at least in triplicate, and each sample set comprised 10 seedlings.

### Confocal microscopy

Seven-day-old seedlings were mounted in a drop of 2% (20 μg/ml) propidium iodide (PI; Sigma-Aldrich) solution, on a microscope slide, and the images were acquired using a LEICA SP5 laser scanning confocal imaging system. The excitation and emission wavelengths are reported in the image captions. High-definition images were acquired (1024×1024, 25× objective) and analysed using the Fiji – ImageJ bundle software (http://fiji.sc/Fiji). The experiments were performed at least in triplicate, and each sample set comprised 10 seedlings.

### Statistics

All experiments were performed at least in triplicate, and the images represent typical examples. The values are represented as the means ± standard deviation. The statistical significance was demonstrated using Student’s *t*-test.

## Results

### *p23-1* and *p23-2* knockout mutants show shorter roots

**Fig. 1.**
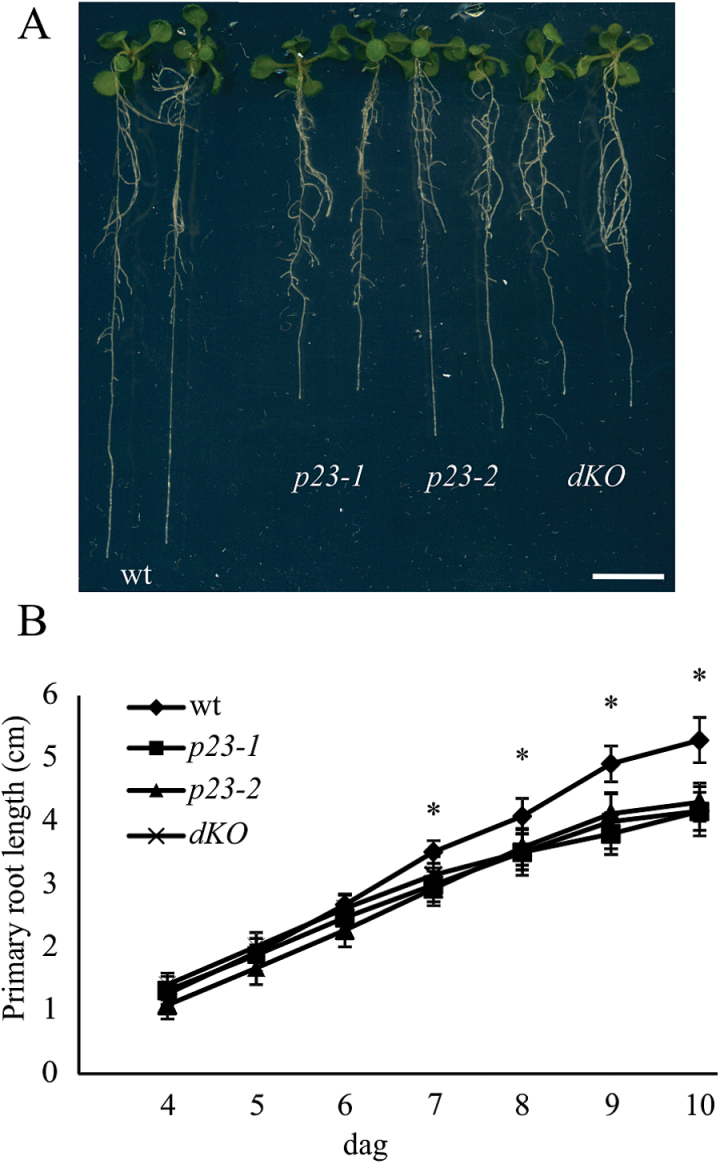
p23 knockout mutants show shorter roots compared with the wild-type. (A) Twelve-day-old *Arabidopsis* seedlings of wild-type (wt), *p23-1*, *p23-2*, and double knockout (*dKO*) background. p23 single knockout mutants and the double knockout mutant show shorter roots as compared with the wild-type. (B) Quantification of the primary root length of the different backgrounds: wt, *p23-1*, *p23-2*, and *dKO* from the fourth to the 10th dag. The short-root phenotype is characterized by a slower root growth appreciable from the seventh dag (*P<0.01, Student’s *t*-test). Scale bar = 1cm. (A colour version of this figure is available at *JXB* online.)

To investigate the functional role of p23 proteins in *Arabidopsis*, knockout mutant lines of the two isoforms were isolated and phenotypically characterized. Although the aerial part of the plant showed no altered traits throughout the plant development, root growth was strongly impaired in single and double (*dKO*) mutants (Fig. 1A). In particular, in 10-d-old single mutant seedlings, the primary root length was approximately 70% that of the wild-type (wt) (wt=5.3±0.36cm; *p23-1*=4.17±0.15cm; and *p23-2*=4.31±0.15cm) (Fig. 1B). In mutant lines, the reduction in root growth was evident and significant from the seventh dag compared with that of the wild-type (Fig. 1B). The short-root phenotype, detectable only at an advance stage of development, suggests that the p23 mutants are likely impaired in meristem maintenance rather than in the early post-embryonic stages of root growth.

Double knockout mutants were generated after crossing *p23-1* and *p23-2* (*dKO*). The latter provides information for determining whether these two proteins play a role in the same pathway controlling root growth and to evaluate the contribution of each isoform to this process. The primary root length of the *dKO* line is comparable with that of both single knockout mutants (*dKO*=4.17±0.2cm), as shown in Fig. 1A and B, thus showing no additive effect of the two mutations. This behaviour suggests that although not redundant, the two proteins intervene in the same process regulating root growth.

### Localization and expression profile of *p23-1* and *p23-2*

**Fig. 2.**
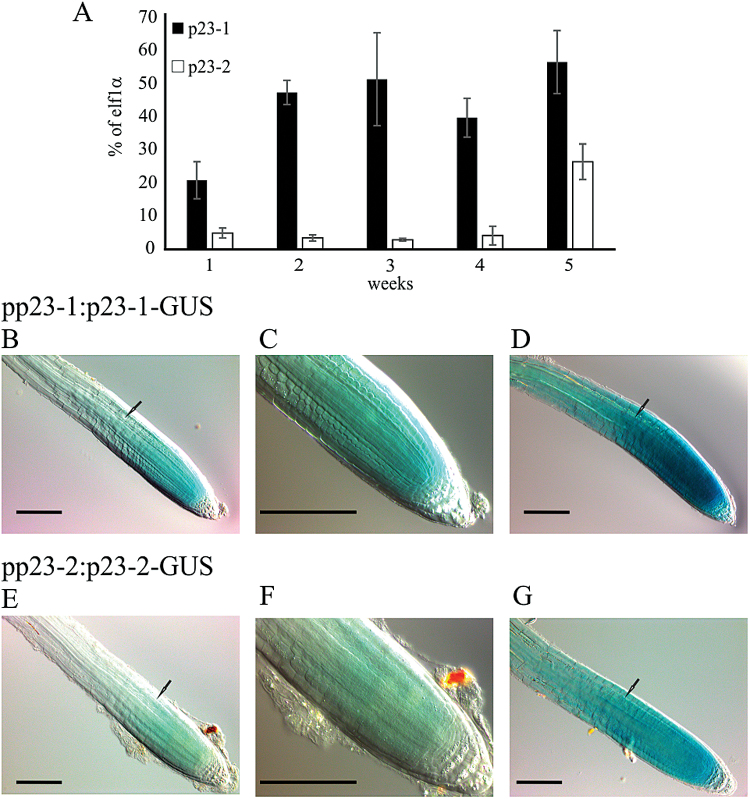
Transcript levels of *p23-1* and *p23-2* and protein localization *in planta*. (A) Quantitative real-time analyses of the transcript levels of *p23-1* and *p23-2* in *Arabidopsis* plants, grown in soil from 1–5 weeks. Data are reported as percentage of *elf1α* transcript levels. (B–G) Histochemical analyses of the translational reporter of p23-1 and p23-2. (B, E) pp23-1:p23-1-GUS and pp23-2:p23-2-GUS *Arabidopsis* staining (30min) concentrates in the root meristem and drops above the first full-elongated cell (arrow). (C, F) Magnification of the root meristem. (D, G) Four hours of GUS staining reveals that both p23 proteins appear also in the upper part of the root. Scale bar = 100 μm. (A colour version of this figure is available at *JXB* online.)

To evaluate the expression profile of the two genes, the transcripts of *p23-1* and *p23-2* were analysed through qRT-PCR during plant development (1–5-week-old plants). As reported in Fig. 2A, both isoforms are expressed at all growth stages. *p23-1* is the most expressed isoform ranging from 20–50% of *elf1α* expression, whereas p23-2 expression ranges from 2–5% of *elf1α* expression. This result demonstrates a 10-fold higher expression of the long isoform than that of the short isoform, during the first 4 weeks and suggests that p23-1, although showing a similar phenotype in the roots, is the major p23 isoform present in plants.

The expression and localization of p23-1 and p23-2 was determined at cellular level *in planta*. The short isoform p23-2 was previously described as nuclear and cytosolic (Tosoni *et al.*, 2011). To analyse the subcellular localization of the long isoform p23-1, stably transformed *Arabidopsis* plants expressing the protein under the control of the constitutive viral promoter CaMV 35S were generated. In these plants, the fusion protein p23-1, tagged at the C-terminus with YFP, was expressed in all tissues. These transgenic lines showed florescence throughout the plant body. The analysis of different plant tissues using confocal laser microscopy defined the subcellular localization of the chimeric protein. p23-1-YFP was detectable in leaves of 10-d-old seedlings (Supplementary Fig. S1 available at *JXB* online) with a cytosolic and nuclear localization and similarly in all analysed tissues.

To examine the expression and localization of the two proteins in the entire plant, stable transformed *Arabidopsis* plants expressing the β-glucuronidase gene (*UID-A*) fused either to *p23-1* or *p23-2* were generated. The reporter allowed defining the localization of the two proteins through a histochemical assay. Several transgenic lines were obtained and seedlings at different developmental stages were analysed.

In 3–10-d-old seedlings, the presence of the chimeric proteins is detectable in the root meristematic zone, where they strongly concentrate (Fig. 2B, C–F). Four hours of staining let the signal appear in the vascular tissue of the roots (Fig. 2D, G) and after 16h the reporter was detected in the entire root and in the vasculature of the leaves (data not shown). It is worthwhile to highlight that the two proteins are highly expressed in the root meristem and the signal drops at the level of the first full-elongated cell (black arrows, Fig. 2B–G). Furthermore, the difference in the expression level of the two isoforms, observed by qRT-PCR (Fig. 2A), was confirmed by the histochemical assay. In fact, although p23-1-GUS is detectable in the root meristem after 5min of staining, p23-2-GUS shows comparable levels only after 30min.

### p23 mutants show a reduced number of dividing cells in the root meristem

**Fig. 3.**
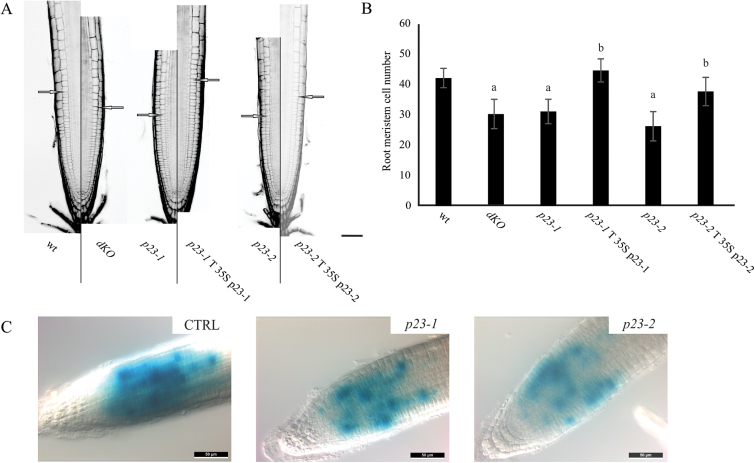
p23 mutant lines show a reduced number of cells in the root meristematic zone. (A) PI stained root of 7-d-old seedlings of the different lines. Excitation and emission wavelengths for confocal acquisition are 488nm and 600–650nm, respectively. The arrows show the upper limit of the meristematic zone. Scale bar = 50 μm. (B) Meristem cell number in the different genetic backgrounds. p23 mutant lines show a reduced number of cortical cells in the root meristematic zone, while complemented lines show no difference as compared with the wild-type (^a^ *P*<0.01 compared with the wt, ^b^ *P*<0.01 compared with the background line; both Student’s *t*-test). (C) Histochemical analysis of the CyclinB1:GUS reporter in the wild-type (CTRL), *p23-1*, or *p23-2* background. Scale bar = 50 μm. (A colour version of this figure is available at *JXB* online.)

The structure of the primary root was accurately analysed in wild-type and mutant lines by confocal microscopy. For this purpose, root width, meristem length, and cell number, in the zone between the quiescent centre and the first cortical elongating cell, were measured (Moubayidin *et al.*, 2013). These analyses revealed that roots of the different genotypes have a comparable width, whereas a significant difference was observed in the length of the meristematic zone (Fig. 3A). As shown in Fig. 3B, both single and *dKO* lines showed a reduced number of cortical cells in the root meristem compared with the wild-type (wt=41.8±3.2; *p23-1.1*=31±4; *p23-2.1*=26±4.8; *dKO*=30±4.8). To verify the causality between the deficiency of p23 proteins and short-root phenotype, stable transformed lines overexpressing p23-1-HA or p23-2-HA were generated in the *p23-1* (*p23-1* T p23-1-HA) or *p23-2* (*p23-2* T p23-2-HA) background, respectively. As reported in Fig. 3A and B, the overexpression of p23 proteins could complement the respective mutant lines both in terms of meristem length and cortical cell number (*p23-1* T p23-1=44.3±3.8; *p23-2* T p23-2=37.40±4.7).

To assess whether the reduction in meristem cell number could reflect the loss of meristematic-cell-division potential, the expression of *pCyclinB1:GUS* (a broadly used marker to indicate the G2‐to‐M phase of the cell cycle) was monitored (Colón-Carmona *et al.*, 1999) in wild-type, and *p23-1* and *p23-2* single mutants, to visualize root meristem cells in the G2–M phase. As shown in Fig. 3C, the *pCyclinB1:GUS* in both *p23-1* and *p23-2* backgrounds showed a strong reduction in the intensity and extent of GUS staining as compared with the wild-type background (CTRL). This result demonstrated that cell division is impaired in the root meristem of both the single mutant lines, further suggesting a common pathway for the two isoforms in regulating root growth.

### Auxin distribution is altered in p23 mutants

**Fig. 4.**
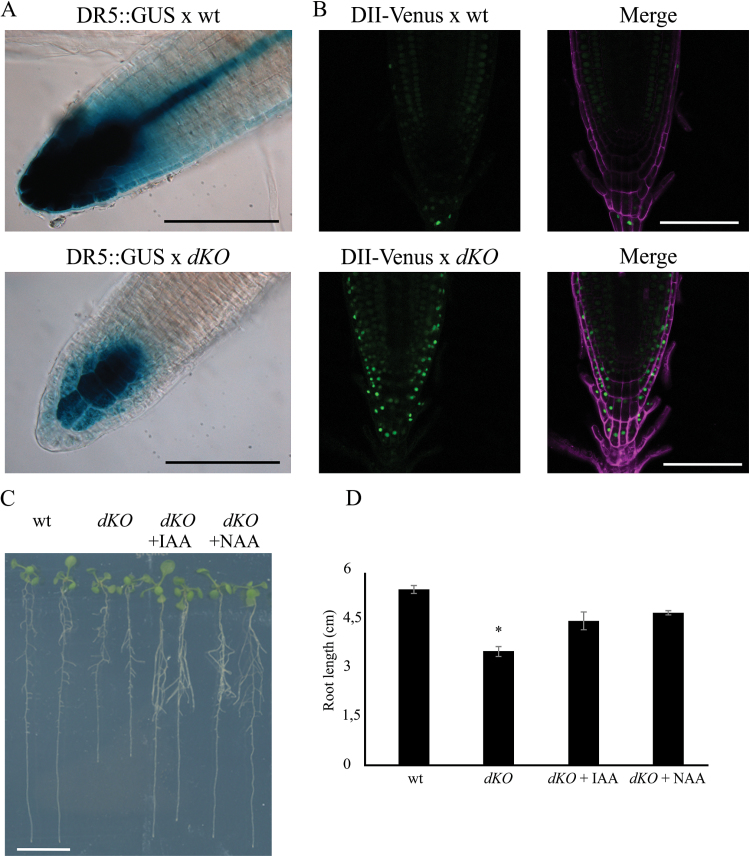
Auxin distribution is altered in the root meristem of the dKO. (A) GUS expression pattern of DR5:GUS in *dKO* background (lower panel) is weaker than in DR5:GUS (upper panel). Scale bar = 100 μm. (B) The green fluorescence of DII-Venus shows much higher levels in the *dKO* background (lower panel) compared with the wild-type (upper panel), in particular in the columella cells and in the epidermis. Venus Ex: 488nm, Em: 520–540nm; PI Ex: 488nm, Em: 600–650nm. Scale bar = 100 μm. (C) *dKO* short-root phenotype is rescued by application of 10nM IAA or NAA to 5-d-old seedlings and analysed after 5 d. Scale bar = 1cm. (D) Quantification of the primary root length of wild-type and *dKO* lines under control condition or upon auxin treatment (*P<0.01, Student’s *t*-test). (A colour version of this figure is available at *JXB* online.)

A p23 double knockout mutant harbouring the DR5::GUS construct was generated to evaluate whether auxin distribution was altered. DR5 is a widely used auxin molecular marker (Ulmasov *et al.*, 1997) that allows the detection of cellular responses to auxin using a histochemical assay. As shown in Fig. 4A, strong DR5 activity was detected in the wild-type primary root (upper panel), particularly at the level of columella cells and the quiescent centre (QC), whereas lower expression was observed in *dKO* plants (lower panel).

To obtain a more detailed analysis of auxin distribution in the meristem at the cellular level, the DII-Venus auxin responsive marker line was crossed with the *dKO* line, leading to a more direct detection system for auxin in the mutant (Brunoud *et al.*, 2012). Analyses of DII-Venus fluorescence using laser scanning confocal microscopy (Fig. 4B) showed that DII-Venus fluorescence was higher in the root meristem of the *dKO* line (lower panels) than in wild-type plants (upper panels). In particular, higher levels of fluorescence were detected in the root cap, epidermis, and columella cells—tissues that are typically characterized by auxin accumulation (Krecek *et al.*, 2009). However, the fluorescent signal was not detectable at the level of the quiescent centre in both wild-type lines, as expected, and p23 mutants, suggesting that this mutation does not compromise auxin levels in the QC. Indeed, the expression of ASB1 (ANTHRANILATE SYNTHASE BETA SUBUNIT 1) and YUC6 (YUCCA 6) in the shoot of *dKO* plants was similar to that of wild-type plants, whereas slightly enhanced expression was observed in the roots (Supplementary Fig. S2 available at *JXB* online). This result suggests that alterations in auxin levels in the roots do not depend on the lower biosynthetic rate of the hormone.

Furthermore, it was observed that in *dKO* mutants treated at 5 dag with 10nM of either IAA or NAA, the wild-type primary root length was rescued (Fig. 4C, D), thus directly associating the short-root phenotype with low auxin content at the root level. These results suggest that alterations in auxin sensing can be excluded. Moreover, both IAA and NAA restored normal root growth, suggesting that the *dKO* line is not altered in auxin influx.

### PINs expression level and localization is altered in p23 double mutants

As auxin sensing and biosynthesis are not the primary cause in determining the reduction of intracellular auxin levels in the *dKO* root, auxin transport was analysed to determine whether this process could cause the short-root phenotype of p23 mutants.

The directed cell-to-cell distribution of auxin is achieved through a system of auxin influx and efflux transporters, and auxin fluxes can be predicted based on the asymmetric plasma membrane distribution of PINs determining PAT (Bailly *et al.*, 2008). PIN transcription is enhanced through auxin and diminished by cytokinin via SHY2 (Dello Ioio *et al.*, 2008). In addition, PIN activity is finely controlled through post-translational modifications, such as phosphorylation by PID (PINOID) and D6PK (D6 PROTEIN KINASE) (Zourelidou *et al.*, 2014), which also control the polar subcellular localization of these proteins (Michniewicz *et al.*, 2007; Marhavý *et al.*, 2014).

**Fig. 5.**
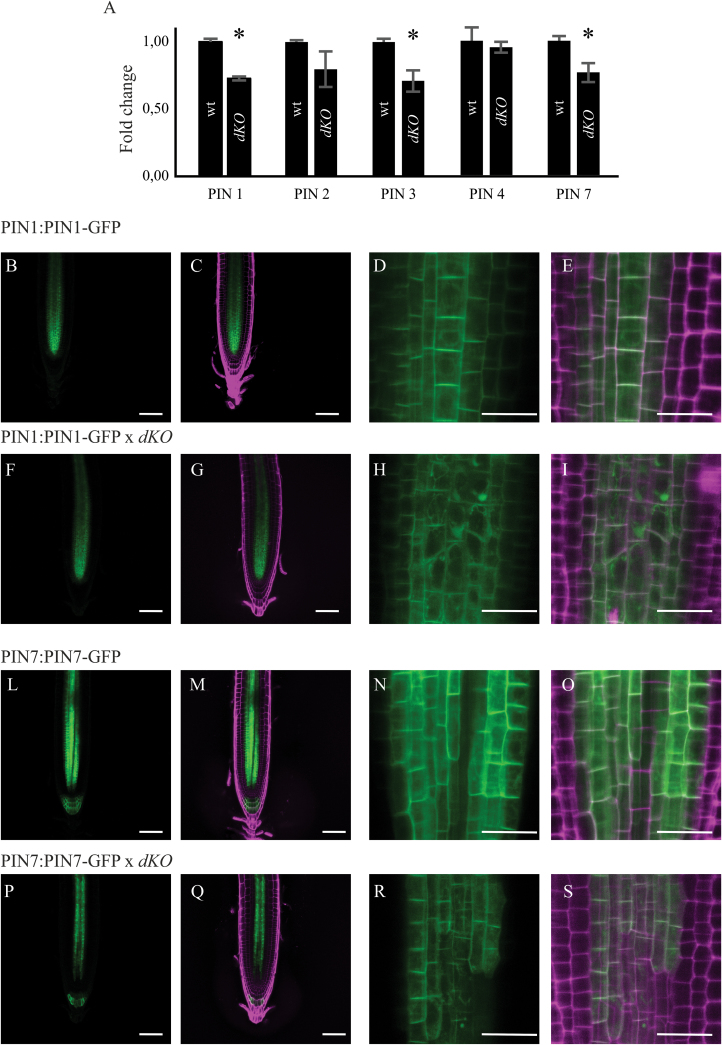
PIN expression levels, and PIN1 and PIN7 localization in the *dKO*. (A) qRT-PCR of PIN1, 2, 3, 4, and 7; data were normalized to ACT2 and shown as fold change on wild-type (*P<0.05, Student’s *t*-test). (B) Confocal Laser Scanning images of PIN1 (B–E), PIN1×*dKO* (F–I) or PIN7 (L–O) and PIN7×*dKO* (P–S) showing altered expression and localization of PIN1 and PIN7 in the mutant line. GFP Ex: 488nm, Em: 500–520nm; PI Ex: 488nm, Em: 600–650nm. Scale bars (B, C, F, G, L, M, P, Q) = 50 μm; (D, E, H, I, N, O, R, S) = 10 μm. (A colour version of this figure is available at *JXB* online.)

The transcription levels of the major PAT protein were assayed, analysing the gene expression level of PIN1, PIN2, PIN3, PIN4, and PIN7 using qRT-PCR analysis. The expression of PIN1, PIN3, and PIN7 was approximately 75% in the *dKO* compared with that of the wild-type (Fig. 5A). PIN proteins undergo regulation at both transcriptional and post-transcriptional levels; thus, the translational reporters of PIN1 and PIN7, the main acropetal auxin transport mediators, were analysed in the *dKO* background. As shown in Fig. 5B, the impaired level of expression of these two carriers is reflected by their translational reporters, as observed in 7-d-old *dKO* seedlings. Particularly, PIN1-GFP and PIN7-GFP reporters confirmed the reduced expression of these proteins in the root tip of *dKO* plants, as appreciable from the quantification of the GFP fluorescence in Supplementary Fig. S3 (available at *JXB* online). In addition, increased PIN1-GFP internalization into subcellular compartments of *dKO* plants was detected compared with that of the wild-type plants (Fig. 5D, E, H, I). However, alterations in the periclinal/anticlinal distribution of PIN1 and PIN7 were not observed in *dKO* mutants. Moreover, PIN7-GFP was absent in the stele cells near the stem cell niche (Fig. 5R, S). These results demonstrate that alterations in PIN expression and localization are indeed the most likely cause of lower auxin levels in p23 mutants, thus linking p23 activity to the maintenance of a correct PAT in the root meristem. In support of this hypothesis, a partial resistance of p23 mutants to treatment with NPA (Supplementary Fig. S4 available at *JXB* online), a well-known inhibitor of PAT (Bailly *et al.*, 2008), was observed. In fact, while low concentration of NPA shortened the primary root of the wild-type, a similar effect was detected in the *dKO* only with NPA concentration >1 μM.

### Rescue of short-root phenotype in the triple mutant *p23-1×* *p23-2×* *arr1-4*

Root meristem maintenance and continuous root growth are guaranteed through a strict equilibrium between cell division and cell differentiation, which is finely controlled through plant hormone crosstalk. In this context cytokinin and auxin play key roles (Dello Ioio *et al.*, 2008; Moubayidin *et al.*, 2010; Overvoorde *et al.*, 2010; Depuydt and Hardtke, 2011; Sankar *et al.*, 2011).

**Fig. 6.**
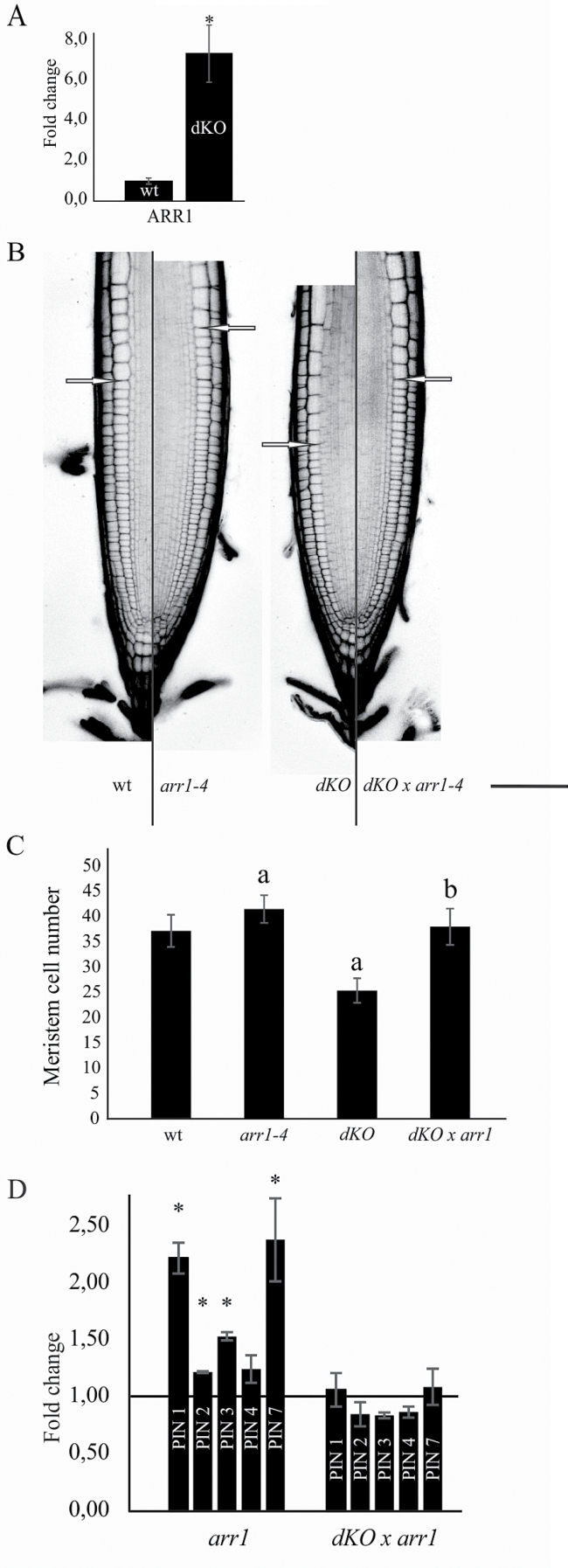
Rescue of short-root phenotype in the triple mutant *p23-1× p23-2×arr1-4*. (A) qRT-PCR of *ARR1*; data were normalized to ACT2 and shown as fold change on wild-type (*P<0.05, Student’s *t*-test). (B) PI-stained root of 7-d-old seedlings of the different lines. Excitation and emission wavelength for confocal acquisition are 488nm and 600–650nm, respectively. The arrows show the upper limit of the meristematic zone. Scale bar = 50 μm. (C) Meristem cell number in the different genetic background. *dKO* mutant shows a reduced number of cortical cells in the root meristematic zone, while *arr1-4* shows an increased number of cortical cells. The triple mutant *dKO×arr1-4* shows no difference as compared with the wild-type (^a^*P*<0.01 compared with the wild-type, ^b^*P*<0.01 compared with the *dKO*; both Student’s *t*-test). (D) qRT-PCR of PIN1, 2, 3, 4, and 7; data were normalized to ACT2 expression and shown as fold change on wild-type (black line) (*P<0.05, Student’s *t*-test). (A colour version of this figure is available at *JXB* online.)

For this reason, the expression of *ARR1*—a key player in cytokinin signalling—was evaluated to test the status of cytokinin signalling in the p23 double knockout mutant. As shown in Fig. 6A, *ARR1* transcripts were markedly upregulated in the *dKO* background compared with wild-type plants (7.4-fold higher). This result suggests that p23 proteins act as negative regulators of the cytokinin signalling pathway, and, at the same time, it supports a role of ARR1 in the short-root phenotype of p23 mutants. To evaluate these hypotheses, the triple mutant line *p23-1×p23-2×arr1-4* (*dKO×arr1-4*) was generated. Laser scanning confocal analysis of the PI-stained root meristem (Fig. 6B, C) showed an average meristem size of 280 µm and an average cortical cell number of 37 in the wild-type line. The p23 *dKO* mutant showed a reduced meristem length and cell number (200 µm and 25 cells, respectively), whereas the single *arr1-4* mutant showed both increased meristem length and meristematic cell number (305 µm and 41 cells), as previously described (Dello Ioio *et al.*, 2007). The triple knockout mutant *dKO×arr1-4* rescued the phenotype of the dKO, showing a meristem length and cortical cell number indistinguishable from those of wild-type (280 µm and 38 cells). In addition, the *arr1-4* mutant was not completely epistatic on the *dKO*, confirming that although restoring a more equilibrated auxin/cytokinin ratio by lowering cytokinin signalling, auxin levels are not sufficient to support the meristem activity present in the single *arr1-4* mutant.

As the *dKO×arr1-4* showed a wild-type-like meristem length, the expression of PINs in this line was tested. As shown in Fig. 6D, the *arr1-4* mutant showed increased expression levels of PIN1, PIN2, PIN3, and PIN7, as previously described (Dello Ioio *et al.*, 2008) and the triple mutant *dKO×arr1-4* recovered PIN expression levels comparable with those of the wild-type. This result supports the prominent role of PIN expression levels in the phenotype of p23 mutants, highlighting, at the same time, an indirect role of p23 proteins on PIN expression.

## Discussion

In the present study, experimental evidence on the role of the p23 co-chaperone as a novel component of the protein network regulating post-embryonic root development has been reported.

Upon seed germination, the root apical meristem grows as cell division prevails over differentiation, reaching a final size at approximately 5 dag, when a fixed meristem cell number is established and meristem maintenance is guaranteed through a balance between the rate of cell differentiation and the rate of new cell generation (Blilou *et al.*, 2005; Dello Ioio *et al.*, 2007, 2008; Moubayidin *et al.*, 2009; Perilli *et al.*, 2012; Pacifici *et al.*, 2015). *Arabidopsis* p23 mutants show impaired root development due to a reduced number of cells in the root meristematic zone, where the two p23 isoforms localize. Using two specific auxin responsive markers (DR5:GUS and DII-Venus), a lower level of auxin accumulation was observed in the root tip of the mutant lines than in the wild-type. It was also shown that the short-root phenotype could be rescued both in single and double knockout mutants by supplying auxin in the growth medium (Supplementary Fig. S5 available at *JXB* online). Based on these results, a constraint at the level of auxin biosynthesis or perception can be excluded, whereas impairment in auxin distribution, reflecting alterations at the level of auxin PAT, was implied and subsequently detected. Indeed, not only were the main components of acropetal PAT—PIN1, PIN3, and PIN7—transcriptionally down-regulated but the localization patterns of PIN1-GFP and PIN7-GFP proteins were also modified. Particularly, PIN1-GFP remains, at least in part, accumulated in intracellular compartments and does not correctly localize to the basal membranes, whereas PIN7 showed a limited localization pattern in the root of the p23 double mutant compared with wild-type. This result suggests that altered PAT is the most likely cause of the reduced meristem length of p23 mutants. In fact, as consequence of the compromised PAT, a narrowed auxin gradient is established in the root meristem of p23 mutants, associated with a shift of the auxin maximum towards the root cap (Grieneisen *et al.*, 2007), together with a reduction in its intensity.

To obtain a more complete picture of hormone signalling regulating meristem maintenance, the expression of *ARR1—*a key component of cytokinin signalling—was evaluated and an increased expression in the mutant compared with wild-type plants was observed. *ARR1*, in turn, is induced through ASB1 (Moubayidin *et al.*, 2013), whose expression was slightly induced in the mutant. These results indicate that cytokinin signalling is enhanced in p23 mutant lines whereas the auxin signalling pathway is reduced. In addition, it was observed that a reduction in cytokinin signalling through the reduction of *ARR1* in the *dKO* mutant generates a wild-type-like phenotype. This triple mutant (*dKO*×*arr1-4*) shows a meristem length and a number of meristematic cortical cells similar to the wild-type, but still reduced compared with that of the *arr1-4* mutant (Dello Ioio *et al.*, 2007), thus revealing the pathway in which p23 operates. Indeed, this result provides genetic evidence that p23 primarily acts on auxin distribution in the root meristem, where these proteins are specifically expressed (Fig. 2B–G). In addition, PIN expression in the triple mutant was tested and PIN levels were found to recover to wild-type values. Taken together, these results confirm that the altered expression and localization of PIN proteins in the p23 double knockout mutant are the primary cause of the reduced meristem length and of the fewer number of meristematic cortical cells. Furthermore, the enhanced level of ARR1 expression could likely be the cause of PINs detriment in p23 mutants, and supports a role of p23 proteins in meristem maintenance a few days after germination, when ARR1 becomes the principal cytokinin effector (Pacifici *et al.*, 2015). At the same time, the recovery of PIN expression level in the triple mutant excludes a direct role of p23 proteins on PIN levels, while its role in PIN trafficking remains to be elucidated. Notably, p23 binds to HSP90 (Zhang *et al.*, 2010), which shows a specific expression at the level of the root (Krishna and Gloor, 2001). Moreover, HSP90 has been demonstrated to interact and regulate TWD1, and consequently ABCB transporters activity, a family of genes involved in extracellular auxin transport that associate with PINs (Pérez-Pérez *et al.*, 2004; Blakeslee *et al.*, 2007; Wang *et al.*, 2013). In this context, the hypothesis is presented that the absence of p23 could compromise the stability of the HSP90 complex, causing alterations in the TWD1-ABCB system, and thus on PAT, leading to reduced auxin levels in the root meristem, altered PIN expression and, as a consequence, a short-root phenotype.

Taken together, these results are consistent with increasing experimental evidence indicating that although cytokinin controls the cell differentiation rate, acting specifically at the transition zone, the graded distribution of auxin is pivotal for the dynamic regulation of root meristem size (Pacifici *et al.*, 2015).

In conclusion, it is proposed that p23 sustains meristem maintenance, playing a key role in post-embryonic root growth via the regulation of auxin signalling and consequently the preservation of a balanced rate of cell differentiation and division at the transition zone.

## Supplementary data

Supplementary data are available at *JXB* online.

Table S1. Primers used in genotyping, cloning, and qRT-PCR.

Figure S1. p23-1-YFP shows nuclear and cytoplasmic subcellular localization.

Figure S2. Auxin biosynthesis is not altered in the shoot of *dKO*.

Figure S3. PIN1-GFP and PIN7-GFP fluorescence quantification in the wild-type and *dKO* backgrounds.

Figure S4. *dKO* is partially insensitive to inhibition of auxin PAT.

Figure S5. Exogenous IAA rescues the short-root phenotype of p23 mutants.

## Funding

This work was supported by the University of Padova [PRAT 2012, CPDA122838] and CARIPARO Foundation to SG.

## Supplementary Material

Supplementary Data
