# Supplementary material for: The co-chaperone p23 controls root development through the modulation of auxin distribution in the Arabidopsis root meristem
Source: J Exp Bot. 2015 Jul 10;66(16):5113–22. doi: 10.1093/jxb/erv330 (PMC4513928; doi:10.1093/jxb/erv330)
Supplement: Supplementary Data [file supp_66_16_5113__index.html]

The co-chaperone p23 controls root development through the modulation of auxin distribution in the Arabidopsis root meristem — The co-chaperone p23 controls root development through the modulation of auxin distribution in the Arabidopsis root meristem — Supplementary Data 

# The co-chaperone p23 controls root development through the modulation of auxin distribution in the *Arabidopsis* root meristem

## Supplementary Data

Data files

- Supplementary Data - Supplementary Data
