# Supplementary material for: The co-chaperone p23 controls root development through the modulation of auxin distribution in the Arabidopsis root meristem
Source: J Exp Bot. 2015 Jul 10;66(16):5113–22. doi: 10.1093/jxb/erv330 (PMC4513928; doi:10.1093/jxb/erv330)
Supplement: Supplementary Data [file supp_erv330_jexbot152637_file001.pdf]

The co-chaperone p23 controls root development through the modulation of auxin distribution in the Arabidopsis root meristem

Stefano D'Alessandro, Serena Golin, Christian S. Hardtke, Fiorella Lo Schiavo, Michela Zottini

Table S1: Primers used in genotyping, cloning and qRT-PCR. List of primers for: cloning of the coding sequences (p23-1-CDS, p23-2-CDS), generating translational reporters (pp23-1:p23-1-GUS, pp23-2:p23-2-GUS), genotyping mutant lines p23-1.1 (SAIL 245_H06; p23-1-Genomic and p23-1-T-DNA) and p23-2.1 (SALK_003076; p23-2-Genomic and p23-2-T-DNA), arr1-4 (ARR1-Genomic and p23-2-T-DNA).

Figure S1: p23-1-YFP show nuclear and cytoplasmic subcellular localization in leaves. Stable transformed *Arabidopsis* lines overexpressing p23-1-YFP show fluorescence in the cytosol and in the nucleus of the cells in leaves of ten-day-old seedlings, YFP Ex: 488 nm, Em: 520-540 nm; PI Ex: 488 nm, Em: 600-650 nm, Chl Ex: 488 nm, Em: 650-750 nm. Scale Bar = 50 μm.

Figure S2: Auxin biosynthesis is not altered in the shoot of *dKO*. Quantitative Real Time analysis of the transcript level of YUCCA6 (YUC6) and ANTHRANILATE SYNTHASE BETA SUBUNIT 1 (ASB1) in seven-day-old *Arabidopsis* seedlings. The expression of the two genes is not altered in the shoot while they resulted slightly up regulated in the root. Data were normalized on ACT2 expression and shown as fold change on the wild type.

Figure S3: PIN1-GFP and PIN7-GFP fluorescence quantitation in the wilt type and *dKO* backgrounds. Image pixel density analysis of PIN1-GFP and PIN7-GFP fluorescence revealed significantly lower levels in the *dKO* background compared to the wild type (* = t test $p < 0.05$). GFP fluorescence (Ex: 488 nm, Em: 500-520 nm) was quantified using the Fiji – ImageJ bundle software. Data are expressed as arbitrary units (A.U.).

Figure S4: *dKO* is partially insensitive to inhibition of auxin polar transport. (A) five-day-old seedlings of wild type and *dKO* backgrounds were treated with different concentrations of NPA. (B)

Primary root length was measured at 10 dag and the *dKO* shows a reduced effect of the treatment on the primary root growth (* = t test $p < 0.05$). Scale Bar = 1 cm.

Figure S5: Exogenous IAA rescues the short root phenotype of p23 mutants. Seedlings of wild type and mutant backgrounds were transferred on 10 nM IAA enriched medium at 5 dag. 10 dag seedlings root length was measured and both single mutants and the double knockout treated with IAA showed longer primary roots compared to control conditions. (a = t test $p < 0.01$ compared to the wt, b = t test $p < 0.05$ compared to *dKO*).

Supplementary Table 1

P23-1-CDS FOR	5’-CATGGAATTCATGAGTCGTCATCCTGA-3’
P23-1-CDS REV	5’-CATGGAATTCCTATGCGTAGTCGGGGACGTCGTAGGGGTA CTTGTCTTCCTTAACAGATG-3’
P23-2-CDS FOR	5’-CATGGGATCCATGAGTCGTAATCCGGAGGTTCTT-3’
P23-2-CDS REV	5’-CATGGAGCTCCTATGCGTAGTCGGGGACGTCGTAGG GGTACTTGTTTCTTGCCTTTTC-3’
pp23-1:p23-1-GUS For	5’-CATGGAATTCACACTCAAAAGTCT-3’
pp23-1:p23-1-GUS Rev	5’-CATGGAATTCCCTTGTCTTCCT-3’
pp23-2:p23-2-GUS For	5’-CATGGGTACCCTCGAGAGCCACACAAGAC-3’
pp23-2:p23-2-GUS Rev	5’-CATGCTCGAGCCCTTGTTTCTTGCCTTTTC-3’
P23-1-Genomic For	5’-TGCATTTTGTTGAGGAACAAAG-3’
P23-1-Genomic Rev	5’-GGAGTCTTTGATTTCTCTGCG-3’
P23-1-T-DNA For	5’-GCCTTTTCAGAAATGGATAAATAGCCTTGCTTCC-3’
P23-2-Genomic For	5’-TCAAGTGGAAAGTAACCATTGC-3’
P23-2-Genomic Rev	5’-AACCGGAAGAGATAGGTGGTC-3’
ARR1-Genomic For	5’-GATCAAACCCATTCAATGTCG-3’
ARR1-Genomic Rev	5’-GAGATGGCATTGTCTCTGCTC-3’
Elf1α qRT For	5’-TGACTCCACCACTGGAGGTT-3’
Elf1α qRT Rev	5’-GGTGGTGGCATCCATCTTGT-3’
P23-1 qRT For	5’-GTCAATGTTGAGGAAAGCAAAATCAAC-3’
P23-1 qRT Rev	5’-CGAGAAATCCATTCCTTCCATTCC-3’
P23-2 qRT For:	5’- AGGTCAACTCTGAAACAGCCT-3’
P23-2 qRT Rev	5’- GGGACGTCGTAGGGGTACTT-3’
PIN1 qRT For	5’- TATTCGGCATCACCTGGTCC-3’
PIN1 qRT Rev	5’- CGCCATGAACAACCCAAGAC-3’
PIN2 qRT For	5’- GCTTCTCATCTCCGAGCAGT-3’
PIN2 qRT Rev	5’- ATCGGTCTGGAGGGGTTCA-3’
PIN3 qRT For	5’- GTGGGAATTCAGTGGCAACG-3’
PIN3 qRT Rev	5’- ATGCGGCCTGAACTATAGCG-3’
PIN4 qRT For	5’- CAACGTGGCAACGGAACAAT-3’

PIN4 qRT Rev	5'- CGATATCATCACCACCACCTCT-3'
PIN7 qRT For	5'- CGTTCATCGGACCAGCTTTG-3'
PIN7 qRT Rev	5'- GGAGCCAATGAACAAGTCGG-3'
ACT2 qRT For	5'- CATTCCAGCAGATGTGGATCTC-3'
ACT2 qRT Rev	5'- ACCCCAGCTTTTTAAGCCTTTG-3'
HSP90.2 qRT For	5'- ACTCACCGTGCTGTCTTGTA-3'
HSP90.2 qRT Rev	5'- GAGGAGAAGCACAAGGTCCT-3'
YUC6 qRT For	5'-AGGTCCACTCGAGCTCAAAA-3'
YUC6 qRT Rev	5'- CCTTCTTATCCCCGAACACA-3'
ASB1 qRT For	5'-GGATTCGTAAGAGGGTTAACGA-3'
ASB1 qRT Rev	5'-GACAATGGCGGCTTCTACAT-3'
ARR1 qRT For	5'-TTTGGTTACCCAGAAGAGATTCA-3'
ARR1 qRT Rev	5'-AGCTCGAACCCAAGAAACAA-3'

Figure S1

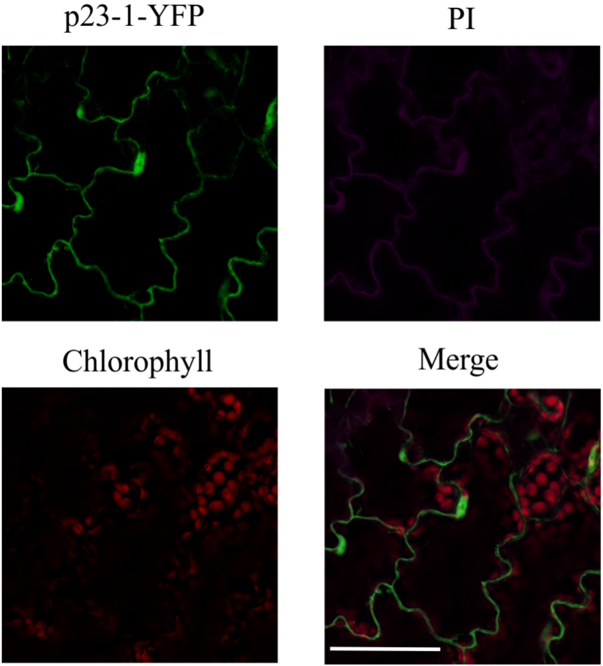

Figure S2

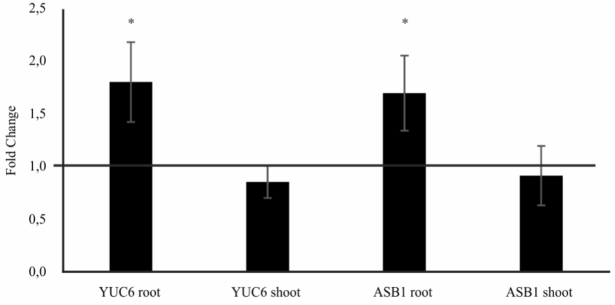

2,5
2,0
1,5
1,0
0,5
0,0
Fold Change
*
*
YUC6 root
YUC6 shoot
ASB1 root
ASB1 shoot

Figure S3

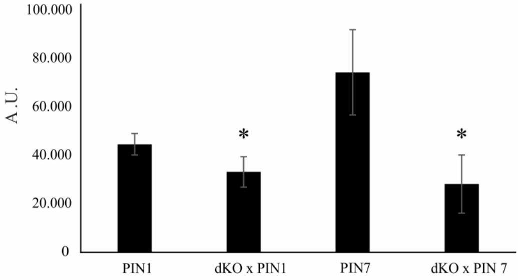
100.000
80.000
60.000
40.000
20.000
0
A.U.
*
*
PIN1
dKO x PIN1
PIN7
dKO x PIN 7

Figure S4

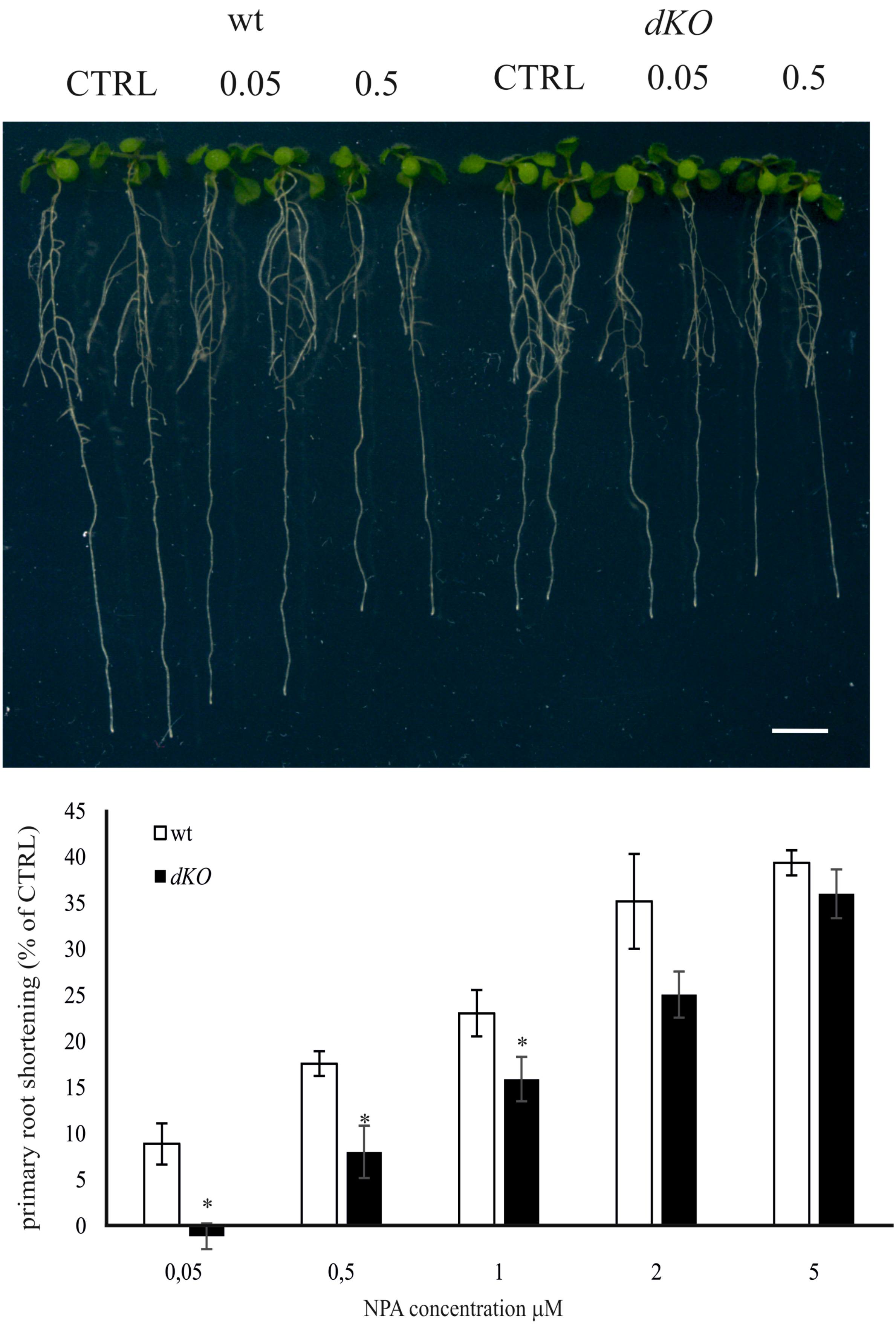

wt
dKO
CTRL
0.05
0.5
CTRL
0.05
0.5
primary root shortening (% of CTRL)
45
40
35
30
25
20
15
10
5
0
wt
dKO
*
*
*
0,05
0,5
1
2
5
NPA concentration µM

Figure S5

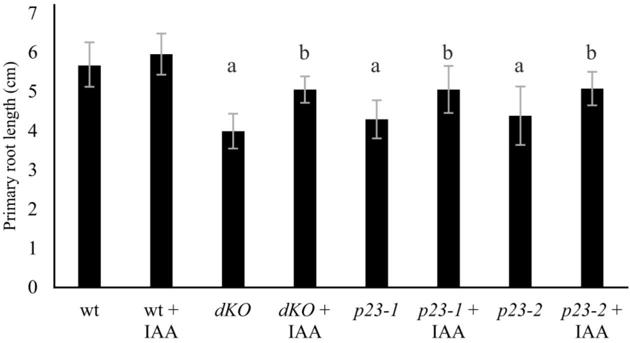

Primary root length (cm)
7
6
5
4
3
2
1
0
a
b
a
b
a
b
wt
wt + IAA
dKO
dKO + IAA
p23-1
p23-1 + IAA
p23-2
p23-2 + IAA
